# Encapsulation-Induced Stress Helps *Saccharomyces cerevisiae* Resist Convertible Lignocellulose Derived Inhibitors

**DOI:** 10.3390/ijms130911881

**Published:** 2012-09-19

**Authors:** Johan O. Westman, Ramesh Babu Manikondu, Carl Johan Franzén, Mohammad J. Taherzadeh

**Affiliations:** 1 School of Engineering, University of Borås, 501 90 Borås, Sweden; E-Mails: rameshmanikondu@hotmail.com (R.B.M.); mohammad.taherzadeh@hb.se (M.J.T.); 2 Chemical and Biological Engineering-Industrial biotechnology, Chalmers University of Technology, 412 96 Göteborg, Sweden; E-Mail: franzen@chalmers.se

**Keywords:** lignocellulosic hydrolysate, ethanol, furfural, HMF (5-hydroxymethyl furfural), carboxylic acids, encapsulation, *Saccharomyces cerevisiae*, biofuel, inhibitors, q-PCR

## Abstract

The ability of macroencapsulated *Saccharomyces cerevisiae* CBS8066 to withstand readily and not readily *in situ* convertible lignocellulose-derived inhibitors was investigated in anaerobic batch cultivations. It was shown that encapsulation increased the tolerance against readily convertible furan aldehyde inhibitors and to dilute acid spruce hydrolysate, but not to organic acid inhibitors that cannot be metabolized anaerobically. Gene expression analysis showed that the protective effect arising from the encapsulation is evident also on the transcriptome level, as the expression of the stress-related genes *YAP1*, *ATR1* and *FLR1* was induced upon encapsulation. The transcript levels were increased due to encapsulation already in the medium without added inhibitors, indicating that the cells sensed low stress level arising from the encapsulation itself. We present a model, where the stress response is induced by nutrient limitation, that this helps the cells to cope with the increased stress added by a toxic medium, and that superficial cells in the capsules degrade convertible inhibitors, alleviating the inhibition for the cells deeper in the capsule.

## 1. Introduction

Second generation biofuels, such as ethanol, are made from lignocellulosic sources, for example agricultural or wood residues. Due to their inherent recalcitrance, such raw materials are not as straightforwardly used as first generation starch-based feedstocks. Either extensive pretreatment followed by enzymatic hydrolysis, or complete acid hydrolysis, is necessary to release the fermentable sugars for fermentation [1]. The pretreatment or acid hydrolysis often creates significant amounts of by-products that act as inhibitors of the subsequent fermentation. Released inhibitors from cellulose and hemicelluloses mainly belong to the three classes carboxylic acids (formic, acetic and levulinic acid), phenolic compounds and furan aldehydes (furfural and HMF) [2–4].

The most widely-used microorganism for industrial fermentations is *Saccharomyces cerevisiae*, since it is capable of producing ethanol at high yields and rates and can also withstand high ethanol concentrations [5]. In order to reach the goal of an effective second generation bioethanol production, it is also of crucial importance to avoid inhibition of the fermentation by the aforementioned inhibitors. Several strains of *S. cerevisiae* are capable of *in situ* detoxification of toxic hydrolysates. However, rather low concentrations of the inhibitors, together with a high concentration of biomass, are required [6]. A lower concentration of inhibitors can be accomplished using fed-batch [6] or continuous cultivations [7], while a higher cell concentration can be achieved by cell immobilization or cell recycling [8,9].

An attractive method of cell immobilization is encapsulation, due to the possibility of achieving cell densities as high as 309 g/L of capsule volume [10]. Macroencapsulated cells are caught inside a gel membrane, within which the cells are suspended in the liquid core. Encapsulating yeast cells not only increases the possible cell concentration in a reactor, but also provides inhibitor resistance. Encapsulated cells have been reported to be able to ferment lignocellulosic hydrolysates that were too toxic for freely suspended cells at the same cell concentration [8]. However, it is not clear why the encapsulated cells are more tolerant, and it is therefore of interest to further study this immobilization system with respect to inhibitor tolerance. One plausible hypothesis is that encapsulated cells are protected by the high local cell density because the superficial cells inside a capsule take care of most inhibitors, letting cells in the core of the capsule experience sub-inhibitory concentrations of the inhibitory compounds. This explanation would require that the cells are able to convert the inhibitors at a relatively high rate.

In order to test this hypothesis, we investigated the effect of encapsulation on the inhibitor tolerance of yeast exposed to two different classes of inhibitors derived from lignocellulosic materials, namely furan aldehydes and weak carboxylic acids. In anaerobic conditions, furan aldehydes are readily converted to less toxic alcohols by yeast [11]. Carboxylic acids are not converted to the same extent under anaerobic conditions, especially in the presence of glucose, since the metabolism of acetic acid is carbon repressed [12]. According to the hypothesis, a medium containing furan aldehyde would be less inhibitory to the encapsulated cells, whereas the fermentability of a medium containing carboxylic acids would not be improved by encapsulation of the fermenting yeast cells. To further characterize the physiological response to encapsulation and the tolerance towards inhibitors, we also investigated the gene expression of the genes *YAP1*, *FLR1* and *ATR1*, known to confer resistance to compounds present in lignocellulosic hydrolysates [13].

## 2. Results and Discussion

### 2.1. Encapsulation Confers Tolerance to Some Inhibitors

Anaerobic cultivations of encapsulated cells were performed in different inhibitory media as well as a non-inhibitory defined glucose medium to investigate the specificity and possible mechanism of the acquired inhibitor tolerance observed in encapsulated yeast. We have previously shown that *S. cerevisiae* CBS8066 was strongly inhibited by both furan aldehydes and carboxylic acids at the same concentrations in the medium as used in the current study, as well as by a dilute acid spruce hydrolysate [14]. The rate of consumption of the first 12 g/L glucose in the media containing carboxylic acid or furan aldehydes was roughly 40% of the rate obtained in the non-inhibitory medium.

**Figure 1:**
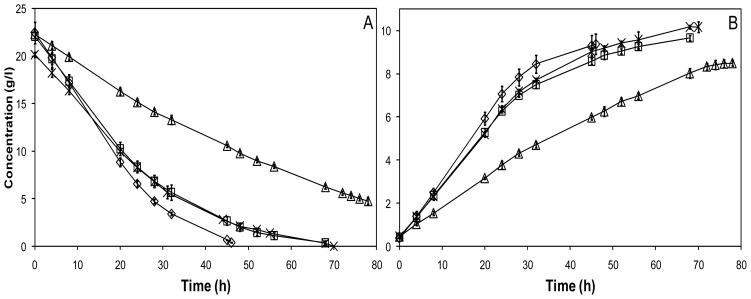
Hexose (**A**) and ethanol (**B**) concentrations during anaerobic batch cultivations using encapsulated yeast in defined glucose medium (DGM) (⋄), DGM with furan aldehydes (□), DGM with carboxylic acids (△), and hydrolysate (×).

Glucose consumption and ethanol production profiles from the anaerobic batch cultivations of encapsulated yeast are presented in Figure 1 and final yields of important metabolites in Table 1. The chitosan-alginate capsules were successful in making the yeast able to ferment the toxic hydrolysate in anaerobic batch cultures (Figure 1). Encapsulation also helped against the mix of furan aldehydes (furfural and HMF), resulting in only slightly slower glucose consumption and ethanol production than what was observed for medium without inhibitors (Figure 1). The consumption rate of the first 12 g/L glucose was approximately 80% of the rate in the non-inhibiting medium. We hypothesize that the high local cell density inside the capsules facilitates a fast conversion of the inhibitors entering the capsule, thus keeping the local inhibitor concentration at a low level. By the end of the cultivations, the overall consumption of furfural and HMF was generally higher for the encapsulated cells than for free cells in similar conditions (Table 2), although the free cells were also able to convert most of the furan aldehydes in the defined medium and some in the hydrolysate medium.

Encapsulation of yeast in chitosan-alginate membranes did not aid in the protection against carboxylic acids inhibitors, showing that the protective effect from encapsulation is specific to some inhibitors. The glucose consumption was markedly slower in medium with carboxylic acid inhibitors than in medium without inhibitors or with furan aldehydes (Figure 1). The consumption rate of the first 12 g/L glucose was close to 40% of that in the non-inhibiting medium, a similar inhibition level to what was seen for the free yeast. The reason for the specificity of the tolerance is likely that the carboxylic acids are not converted to less inhibitory compounds to the same extent as the furan aldehydes. A high local cell density yeast population does thus not help to keep the concentrations of carboxylic acids at a lower local level inside the capsules.

The biomass yield was significantly lower for the encapsulated cells compared to the free cells in all media tested, with no apparent cell growth in the carboxylic acids medium for the encapsulated cells. The lower biomass yield is probably an effect of the low level of budding cells inside the capsules [15], where more cells are likely consuming glucose only for maintenance energy. The glycerol yields of the encapsulated yeast were generally lower, whereas the acetate yields were higher, in yeast grown in inhibitory media compared to the non-inhibitory medium, similar to previously observed trends [16]. The acetate yield was, in most cases, also lower for the encapsulated yeast, whereas the glycerol yield seemed to be generally higher for the encapsulated yeast when compared to the free yeast under the conditions tested.

### 2.2. Encapsulation Triggers Stress Responses

In order to further investigate the protective effect of encapsulation, the expression of three relevant stress-responsive genes was quantified by q-PCR for cells grown in the different media. The genes chosen for expression analysis have been investigated in an over-expression study where they were shown to confer resistance to lignocellulose-derived inhibitors [13]. *YAP1* encodes a transcription factor and responds to various different stress conditions. It confers resistance to coniferyl aldehyde, HMF and spruce hydrolysate [13]. It has also been shown to activate pleiotropic drug resistance [17] and to be important in oxidative stress response [18] as well as in the response to carbon stress [19]. *YAP1* is also involved in the control of *ATR1* [20] and *FLR1* [21], which encode membrane transport proteins required for aminotriazole [22] and fluconazole [21] resistance, respectively. When over-expressed, *ATR1* has also been shown to confer resistance to coniferyl aldehyde, and *FLR1* to both coniferyl aldehyde and HMF, common inhibitors in spruce hydrolysate [13].

**Figure 2:**
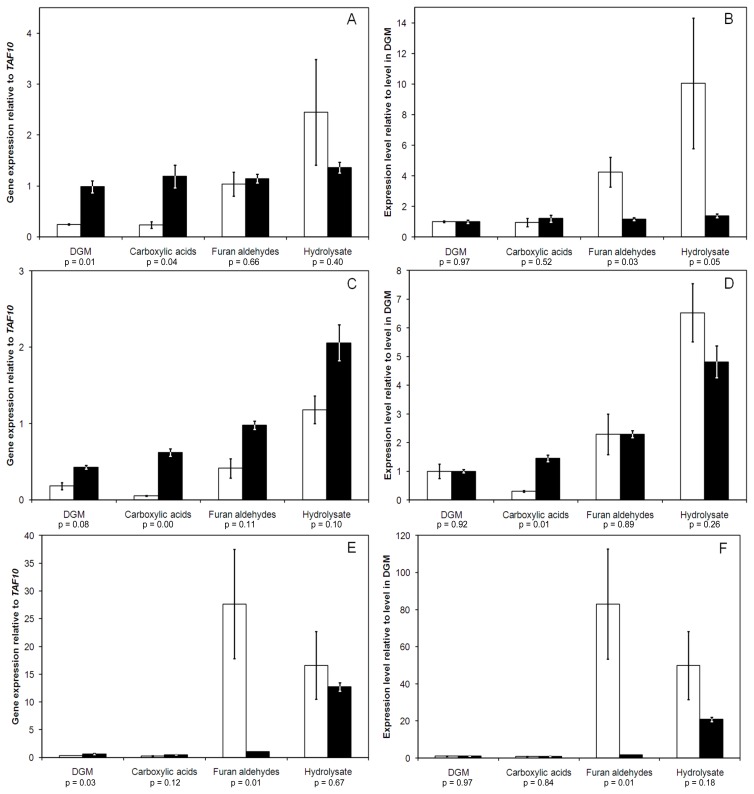
Gene expression in different media after two hours of anaerobic batch cultivations; (**A**, **C** and **E**) *YAP1*, *ATR1* and *FLR1* respectively, relative to the level of *TAF10*, (**B**, **D** and **F**) *YAP1*, *ATR1* and *FLR1* respectively, relative to the expression level in DGM, for free yeast (white) and encapsulated yeast (black). The error bars depict variation in two biological replicates. The equality of means was tested using the logarithm of the relative gene expression in two-sided *t*-tests, assuming equal variance. Observed *p*-values are shown under each pair (Data from free yeast is from [14]).

The gene expression analysis supported the finding that the cells inside the capsules grown in furan aldehydes medium and hydrolysate were less stressed, or at least found the changes in stress level less challenging, compared to freely grown cells under similar conditions. The expression of the transcription factor *YAP1* was relatively unchanged in the encapsulated cells in different media, with only a slightly higher level in hydrolysate medium (Figure 2A,B). However, the *YAP1* expression level was increased due to encapsulation already in the medium without inhibitors (Figure 2A), indicating that the cells sense a stress as a direct or indirect consequence of being encapsulated. This could have been beneficial when transferred to an inhibitory medium since they, through the activation of the initial stress response, may be better prepared to cope with the increased stress posed by the inhibitors. The expression levels of *ATR1* were higher in the encapsulated cells than in the free cells for all media (Figure 2C). However, the relative change in expression from the level in DGM was higher only in the case of carboxylic acids, against which the encapsulation did not provide significant protection (Figure 2D). The most prominent change due to the encapsulation was found in the expression of *FLR1* in furan aldehydes medium (Figure 2E,F). The expression was only slightly increased in the encapsulated cells, whereas a large increase in expression was observed for the free cells. Also for this gene, the expression relative to *TAF10* was higher in DGM in the encapsulated than in the free cells (Figure 2E), leading to less drastic increases in the encapsulated cells when subjected to inhibitory media (Figure 2F).

The increased levels of stress-related genes in non-inhibitory medium could arise from a minor starvation of cells close to the center of the capsules, as there are probably nutrient limitations due to mass transfer limitations. Ge *et al*. [23] showed that intra-particle mass transfer limitations arise in flocs larger than 100 μm. Thus, it is obvious that mass transfer limitations are present in a capsule of 3–4 mm in diameter with yeast inside.

**Figure 3:**
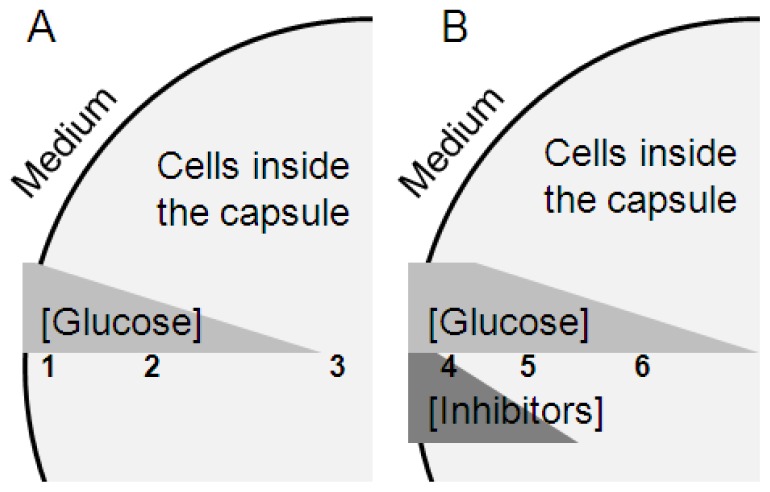
Schematic figure of the hypothesized glucose and inhibitor concentration profiles in a cross section of the capsules in non-inhibitory medium (**A**) and medium with convertible inhibitors (**B**) and the corresponding differences in cell physiology. The numbers indicate different cell populations: 1. non starved cells; 2. slightly starvation-stressed cells with triggered ESR; 3. starved cells; 4. non-growing cells converting inhibitors; 5. ESR triggered cells, growing and converting inhibitors; 6. slightly starvation-stressed cells.

Due to metabolism and mass transfer limitation, it can be visualized that the physiology of the cells inside a capsule changes, depending on their position along the radius of the cell pellet. In a non-inhibitory medium, superficial cells thrive while the interior cells suffer from nutrient limitation due to both mass transfer limitation and consumption, giving rise to a slight stress response. In a medium with readily convertible inhibitors, on the other hand, the cells close to the surface are forced to convert the inhibitors, leaving glucose for cells deeper in the cell pellet. Depending on the outer conditions, in this way there will be different parts of the capsule where cells thrive, as schematically depicted in Figure 3.

In the gene expression analysis, we see a mean value of all cells in the capsules, why cells at different depth inside the capsules (Figure 3) are likely to have higher or lower expression levels than the reported mean values. It has previously been shown that slowly growing yeast has an increased stress resistance [24] and this could be one reason for the increased inhibitor tolerance of encapsulated yeast cells as part of the Environmental Stress Response (ESR) [25]. The already slightly stressed cells would be helped to easier adapt to the new situation of inhibitors being present in the growth medium [26], whereas the cells closest to the surface would be sacrificed. This implies that the improved tolerance to lignocellulose-derived inhibitors is not simply a result of less inhibitors coming in contact with the cells when they are encapsulated, but also that at least some cells are actually better prepared to handle them. However, the encapsulated cells still require a means of reducing the levels of inhibitors by converting them to less inhibitory compounds.

### 2.3. Diffusion Rate through the Capsule Membrane Is Enhanced by Chitosan

**Figure 4:**
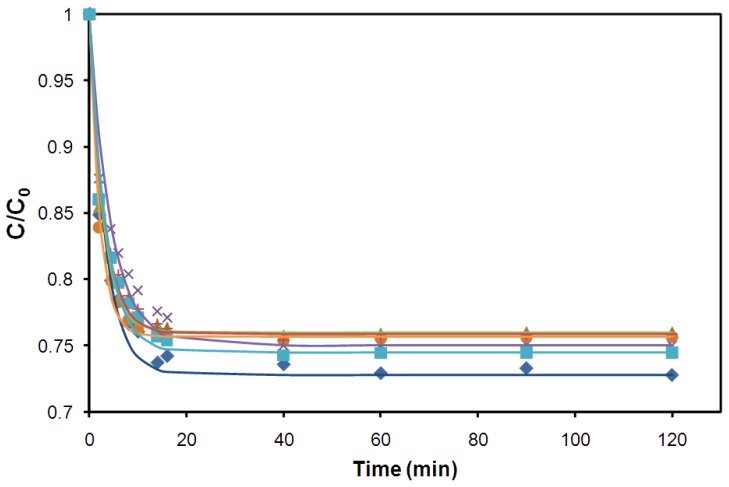
Time course of diffusion through the membrane of the chitosan-alginate capsules; glucose ( 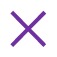), HMF ( 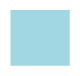), furfural ( 
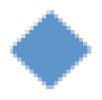
), levulinic acid ( 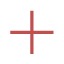), acetic acid ( 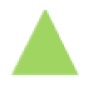) and formic acid ( 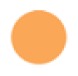). The lines show the profile of diffusion according to Equation 1 for the respective compounds.

A possible explanation for the observed differences in tolerance towards the furan aldehyde and carboxylic acid inhibitors could be differences in their diffusivity into the capsules. This was the case for the hydrophobic inhibitor limonene, which could not diffuse into the capsules [27]. The diffusion of glucose and the inhibitors furfural, HMF, acetic acid, formic acid and levulinic acid into the capsules was therefore investigated. The capsules used in the test did not contain yeast, and were equilibrated in water prior to the test. The diffusion of the compounds into the capsules was monitored for 120 min (Figure 4), after which water was added and the diffusion out of the capsules was monitored. Although the acids had a somewhat faster diffusion into the capsules than the furan aldehydes (Table 3), it can most likely be ruled out as a reason for the increased tolerance towards furan aldehydes, since the diffusion was rather fast for all tested compounds. The diffusion rate was markedly faster than what has previously been reported for Ca-alginate capsules, identical to the capsules used in this study, except for the additional chitosan incorporation [8,15]. The results show that the chitosan treatment of the capsules resulted in a faster diffusion of glucose, HMF, furfural and acetate, with 95 ± 3% of the final concentrations already after 14 min, compared to the reported 90 ± 3% after 20 min without chitosan treatment [8]. The volumetric mass transfer coefficient for glucose into the chitosan-alginate capsules was calculated to 10.04 (cm^3^/min), which can be compared with a value of 6.28 (cm^3^/min) calculated for cell-free Ca-alginate capsules in a similar experimental setup [15]. The volumetric mass transfer coefficients for the compounds tested was proportional to the molecular weight of the compound (Table 3), with lighter molecules diffusing faster into the capsules. The diffusion out of the capsules was slightly slower than the diffusion in, (96 ± 3% of the final concentrations after 20 min), likely due to the slower internal mixing rate inside the capsules. The tendency towards an over-estimation of the glucose concentration at early time points by Equation 1, both in Figure 4 and a previous work [15], is hypothesized to come from osmosis of water from the capsules to the surrounding solution, because of the high solute concentration, shrinking the capsules (also noticed by visual observation), and thus lowering the measured solute levels. As the solute concentrations are leveled out, the capsules regain their original shape.

## 3. Experimental Section

### 3.1. Yeast Strains and Medium

The *S. cerevisiae* CBS8066 obtained from Centraalbureau voor Schimmelcultures (Delft, The Netherlands) was used in all experiments. It was maintained on YPD agar plates (10 g/L of yeast extract (Scharlau), 20 g/L of soy peptone (Fluka), 20 g/L agar (Scharlau) and 20 g/L of d-glucose (Fisher Scientific) as an additional carbon source).

The growth medium used for the batch cultivations was a defined glucose medium (DGM), as previously reported [28], with glucose as energy and carbon source. The compositions of media for the batch cultivations were a complete spruce hydrolysate as well as DGM (20 g/L glucose) with and without addition of various inhibitors in the following concentrations: the furan aldehydes medium contained 2 g/L 5-hydroxymethyl furfural (HMF) and 1.5 g/L furfural and the carboxylic acids medium contained 200 mM each of acetic, formic and levulinic acid. The hydrolysate contained, according to what was identified using HPLC, glucose 9.2 ± 0.2 g/L, mannose 12.5 ± 0.2 g/L, galactose 2.5 ± 0.0 g/L, xylose 5.2 ± 0.1 g/L, arabinose 1.7 ± 0.0 g/L, acetic acid 2.2 ± 0.1 g/L, furfural 0.19 ± 0.02 g/L, HMF 0.79 ± 0.01 g/L, catechol 0.03 ± 0.01 g/L and vanillin 0.08 ± 0.01 g/L after supplementation of salts, trace metals and vitamins at the same concentrations as in the DGM.

### 3.2. Encapsulation Procedure

The capsules were prepared by the liquid-droplet-forming method [29]. Yeast cells were grown in 250 mL cotton-plugged Erlenmeyer flasks with 100 mL DGM for 24 h in a water bath at 30 °C at 130 rpm. Yeast from 50 mL of the cultivation was harvested at 3500 g for 5 min and resuspended in 50 mL 1.3% (*w*/*v*) sterile CaCl_2_ solution containing 1.3% (*w*/*v*) carboxymethylcellulose (CMC) (Product number 419303, Sigma-Aldrich) with average Mw of 250 kDa and degree of substitution 0.9. CMC increases the viscosity of the CaCl_2_ solution, thereby enhancing the formation of spherical capsules. A sterile solution containing 0.6% (*w*/*v*) sodium alginate (Product number 71238, Sigma) and 0.1% (*v*/*v*) Tween 20 (Sigma-Aldrich) was used for capsule formation. The surfactant Tween 20 improves the permeability of the capsule membrane, thereby preventing the capsules from rupturing due to CO_2_ formation during the cultivation [30]. Capsules were formed by dripping the CMC-yeast-CaCl_2_ solution into the stirred sodium alginate solution through syringe needles (21 G × 1.5 inches). The capsules were gelled for 10 min, washed with distilled water and hardened in 1.3% (*w*/*v*) CaCl_2_ solution for 20 min. The Ca-alginate capsules were thereafter treated with a 0.2% (*w*/*v*) low molecular weight chitosan (Product number 448869, Aldrich) solution with 300 mM CaCl_2_ in 0.040 M acetate buffer, pH 4.5, at a ratio of 1:5 of capsules to solution, for approximately 24 h. The treatment was performed in 1 L Erlenmeyer flasks in a water bath at 30 °C at 140 rpm. Chitosan molecules were incorporated in the alginate matrix, thereby improving the strength of the capsule membrane [31]. After washing the capsules in 0.9% NaCl solution, yeast in 10 mL capsules was cultivated aerobically in 100 mL DGM (50 g/L glucose), which was changed after 24 h to fresh medium for another 24 h for cell propagation prior to the anaerobic batch cultivations.

### 3.3. Batch Cultivations

The batch cultivations were carried out in 250 mL conical flasks, cotton plugged for aerobic cultivation and equipped with rubber stoppers with two stainless steel capillaries and a loop trap for anaerobic cultivations as previously described [32], however without purging with N_2_ (g) prior to cultivation. Sterile water was used in the loop traps to permit produced CO_2_ to escape from the flasks. The anaerobic cultivations were started with 50 capsules in 120 mL fresh medium of different composition. Samples for HPLC analyses were taken through one of the steel capillaries. The samples were centrifuged to remove possible particles and cells and stored at −20 °C until analysis. Biomass samples were only taken at the beginning and the end of each batch, due to the difficulties of taking these samples during the cultivations.

### 3.4. Quantitative PCR

Cell samples to study the expression of *YAP1*, *FLR1*, and *ATR1* were taken after two hours of anaerobic cultivation with inhibitors as described in Section 2.3. The capsules were broken and the cells washed with ice cold water. The samples were thereafter centrifuged for 1 min at 10,000× *g* in a microcentrifuge, the pellet was frozen in N_2_ (l) and thereafter stored at −80 °C until RNA extraction. RNA extraction, q-PCR analysis and data evaluation were performed as previously described [14]. The primer sequences used in the analysis were designed from the sequences listed in the *Saccharomyces* Genome Database (http://www.yeastgenome.org/). The primer sequences used are found in Table 4. The expression levels of the resistance genes were normalized to the expression of the internal reference gene *TAF10* that showed a stable expression in all samples. *TAF10* has previously been shown to be a suitable reference gene for q-PCR experiments with *S. cerevisiae* [33]. The levels were also normalized to the expression level in defined glucose medium, for easier comparison.

### 3.5. Analytical Methods

The amounts of metabolites and inhibitors were quantified by HPLC using an Aminex HPX-87H column (Bio-Rad, Hercules, CA, USA) at 60 °C with 5 mM H_2_SO_4_ as eluent at a flow rate of 0.6 mL/min. A refractive index detector (Waters 2410, Milford, MA, USA) was used for glucose, glycerol and ethanol and a UV absorbance detector (Waters) was used for formic acid, acetic acid, levulinic acid, furfural and HMF. For the hydrolysate samples, an Aminex HPX-87P (Bio-Rad) column at 85 °C with ultrapure water as eluent at a flow rate of 0.6 mL/min was used to analyze the glucose, xylose, galactose and mannose concentrations. These compounds were detected using a refractive index detector (Waters 2410, Milford, MA, USA).

The cell dry weight was measured in pre-dried and pre-weighed glass tubes or watch glasses. Cells from 10 capsules were washed out with distilled water after mechanical crushing of the capsules. The cells were washed once with distilled water before drying for approximately 24 h at 105 °C.

### 3.6. Statistics, Yields and Elemental-Balance Calculations

The biomass and metabolite yields were calculated from the determined concentrations at the end of the fermentations. Error intervals shown are ±95% confidence intervals of the mean unless otherwise mentioned.

### 3.7. Diffusion through the Capsule Membrane

The diffusion into the capsules was tested using 60 mL of cell-free capsules that were added to 180 mL of a solution containing glucose, furfural, HMF, acetic acid, levulinic acid and formic acid at the same concentrations used in the anaerobic batch cultivations as described above. Samples were thereafter withdrawn from the bulk solution at time intervals and the decreasing concentrations were analyzed using HPLC as described above. The diffusion out of the capsules was tested by addition of 100 mL ultrapure water to the solution after equilibrium was reached and the increasing concentrations of the solutes were analyzed using HPLC.

Determination of the mass transfer coefficient was performed as described elsewhere [15]. In short, assuming there are no concentration gradients inside the capsule and a capsule to solute volume ratio of 1:3, the concentration ratio can be expressed as:

(1) $$\frac{{\text{C}}_{\text{t}}}{{\text{C}}_{0}}=\frac{{\text{C}}_{\text{eq}}}{{\text{C}}_{0}}+\;(1-\frac{{\text{C}}_{\text{eq}}}{{\text{C}}_{0}})\;{\text{e}}^{-4\frac{{\text{K}}_{\text{t}}}{{\text{V}}_{0}}}$$

Where *C*_0_, *C_eq_* and *C_t_* are the initial, equilibrium and time *t* concentrations of solute in the solution outside the capsules respectively, and *V*_0_ is the volume of the solution outside the capsules. Estimation of the volumetric mass transfer coefficient, *K* (cm^3^/min), was performed by fitting the theoretical curve from Equation 1 to experimental data with the nonlinear regression function SOLVER in Excel (Microsoft, Redmond, WA, USA) according to [34].

## 4. Conclusions

This study shows that encapsulation enhances tolerance towards certain lignocellulose-derived inhibitors. The high local cell density inside the capsule forces the cells close to the membrane of the capsule to take care of readily convertible inhibitors such as furan aldehydes, letting cells in the core of the capsule experience sub-inhibitory levels, ensuring survival of the population as a whole. Encapsulation does thus not aid significantly against the inhibitory effect of non- or less convertible compounds, such as weak acids in anaerobic conditions. It was further shown that the protective effect could be observed also on a transcript level, with higher expression levels of the stress-related genes in non-inhibitory medium in encapsulated cells as compared to free cells. Due to the higher initial level of transcripts important for inhibitor resistance, a lower relative increase of the genes in inhibitory media containing furan aldehydes could be observed, helping the cells to cope with the increased stress. The higher tolerance towards furan aldehydes compared to acids was not due to exclusion of furan aldehydes from the capsules, since all inhibitors easily diffused into the capsules. We also showed that the alginate capsules treated with chitosan facilitated a faster diffusion of glucose than was reported for non-treated capsules. A faster diffusion of glucose into the capsules lowers the impact of mass transfer limitations, likely leading to higher maximum ethanol production rates.

## Figures and Tables

**Table 1:** Final yields on consumed hexoses in anaerobic batch cultivations.

Medium	Cultivation mode	Y_SE_	Y_SAce_	Y_SGly_	Y_SBiomass_
Defined glucose medium (DGM)	Enc.	415 ± 30	1 ± 2	57 ± 5	51 ± 8
	Free	435 ± 10	11 ± 3	44 ± 2	64 ± 4

Carboxylic acids	Enc.	462 ± 4	18 ± 4	25 ± 6	−2 ± 2
	Free	416 ± 48	32 ± 10	73 ± 8	13 ± 7

Furan aldehydes	Enc.	426 ± 9	30 ± 3	41 ± 2	24 ± 1
	Free	432 ± 12	8 ± 2	33 ± 2	30 ± 3

Hydrolysate	Enc.	484 ± 23	7 ± 10	54 ± 6	34 ± 1
	Free	411 ± 5	70 ± 26	24 ± 10	76 ± 37

Yields are shown as 95% confidence intervals (*n* ≥ 2) in mg product per g consumed hexose. Enc.: Encapsulated CBS8066; Free: Free CBS8066 (from [14]); Y_SE_: Ethanol yield; Y_SAce_: Acetate yield; Y_SGly_: Glycerol yield; Y_SBiomass_: Biomass yield.

**Table 2:** Overall inhibitor conversion in anaerobic batch cultivations.

Medium	Cultivation mode	HMF (%)	Furfural (%)
Furan aldehydes	Enc.	61 ± 3	100 ± 0
	Free	73 ± 2	100 ± 0

Hydrolysate	Enc.	74 ± 2	99 ± 1
	Free	10 ± 2	67 ± 11

Initial concentrations of the furan aldehydes; 2.0 and 0.79 g/L 5-hydroxymethyl furfural (HMF) and 1.5 and 0.19 g/L furfural in the furan aldehydes medium and hydrolysate respectively. The decrease is shown as the percentage removed from the initial concentration of the inhibitory compound, with 95% confidence intervals (*n* ≥ 2) of free [14], and encapsulated (Enc.) *S. cerevisiae* CBS8066 grown in the indicated media.

**Table 3:** Volumetric mass transfer coefficient into empty chitosan-alginate capsules.

Compound	K (cm^3^ min^−1^)	Mw (g mol^−1^)
Formic acid	20.69	46.03
Acetic acid	15.99	60.05
Levulinic acid	14.65	116.11
Furfural	13.44	96.08
HMF	13.13	126.11
Glucose	10.04	180.16

**Table 4:** Primer sequences used in the q-PCR analysis.

Gene	Forward (5′→3′)	Reverse (5′→3′)
*ATR1*	ATTCTTTGGATGGGGCTCTT	AGCCCACATTGAATGCTACC
*FLR1*	GCCTGCCTCTGTCTTTGTTC	ACCAAACAACGGAAAAGCAC
*YAP1*	TACACGTGATGGCGAGGATA	CCACTTCATTTTGCTGCTGA
*TAF10*	TACCCGAATTTACAAGAAAAGATAAGA	ATTTCTGAGTAGCAAGTGCTAAAAGTC
